# Dietary Indole-3-Carbinol Alleviated Spleen Enlargement, Enhanced IgG Response in C3H/HeN Mice Infected with *Citrobacter rodentium*

**DOI:** 10.3390/nu12103148

**Published:** 2020-10-15

**Authors:** Yanbei Wu, Jing Wang, Qiang He, Liangli Yu, Quynhchi Pham, Lumei Cheung, Zhi Zhang, Young S. Kim, Allen D. Smith, Thomas T. Y. Wang

**Affiliations:** 1 China-Canada Joint Lab of Food Nutrition and Health (Beijing), Beijing Technology & Business University, Beijing 100048, China; yanbeiwu@btbu.edu.cn (Y.W.); wangjing@th.btbu.edu.cn (J.W.); 2 Diet, Genomics, and Immunology Laboratory, Beltsville Human Nutrition Research Center, USDA-ARS, Beltsville, MD 20705, USA; quynhchi.pham@usda.gov (Q.P.); lumei.cheung@usda.gov (L.C.); 3 Department of Nutrition and Food Science, University of Maryland, College Park, MD 20742, USA; lyu5@umd.edu (L.Y.); zzhangkk@terpmail.umd.edu (Z.Z.); 4 College of Biomass Science and Engineering, Sichuan University, Chengdu 610065, China; heq361@163.com; 5 Nutritional Science Research Group, Division of Cancer Prevention, National Cancer Institute, National Institutes of Health, Bethesda, MD 20892, USA; kimyoung@mail.nih.gov

**Keywords:** indole-3-carbinol, C3H/HeN mice, *Citrobacter rodentium*, colitis, immunoregulation

## Abstract

Enteropathogenic and enterohemorrhagic *Escherichia coli* are important enteric pathogens that induce hemorrhagic colitis or even fatal hemolytic uremic syndrome. Emerging evidence shows that some bio-actives derived from fruits and vegetables may serve as alternatives to antibiotics for overcoming multidrug resistant *E. coli* infections. In this study, the *Citrobacter rodentium* (Cr) infection model was utilized to mimic *E. coli*-induced acute intestinal inflammation, and the effects of a cruciferous vegetable-derived cancer protective compound, indole-3-carbinol (I3C), on the immune responses of Cr-susceptible C3H/HeN mice were investigated. Dietary I3C significantly inhibited the loss of body weight and the increase in spleen size in Cr infected mice. In addition, I3C treatment reduced the inflammatory response to Cr infection by maintaining anti-inflammatory cytokine IL-22 mRNA levels while reducing expression of other pro-inflammatory cytokines including IL17A, IL6, IL1β, TNF-α, and IFN-γ. Moreover, the serum cytokine levels of IL17, TNF-α, IL12p70, and G-CSF also were down-regulated by I3C in Cr-infected mice. Additionally, dietary I3C specifically enhanced the Cr-specific IgG response to Cr infection. In general, dietary I3C reduced the Cr-induced pro-inflammatory response in susceptible C3H/HeN mice and alleviated the physiological changes and tissue damage induced by Cr infection but not Cr colonization.

## 1. Introduction

Enteropathogenic and enterohemorrhagic *Escherichia coli* (EPEC and EHEC) are some of the most common pathogenic bacteria that contribute to clinical infections in both animals and humans [1]. EPEC/EHEC exist as normal microbes in the gastrointestinal tract of ruminant animals, such as cow and sheep, but are asymptomatic, while causing severe disease in humans [2]. The symptoms in humans include abdominal cramps, vomiting, and/or diarrhea, which may progress to hemorrhagic colitis [3,4]. About 30% of confirmed cases require hospitalization, and about 10% of cases develop into hemolytic uremic syndrome (HUS) that is characterized by anemia, kidney failure, and low platelet counts [5,6]. Antibiotics are commonly prescribed for bacterial infections but their overuse on farms and in hospitals has increased the incidence of multidrug resistant (MDR) *E. coli* infections, which have become a significant threat to human health [7]. The development of alternative strategies for controlling the spread and treatment of EPEC and EHEC are necessary to protect public health and minimize the economic costs associated with outbreaks of these bacteria.

The study of EPEC and EHEC is limited by the inability of these bacteria to mimic human infections and disease when using a rodent model. *Citrobacter rodentium* (Cr) is a gram negative microbe that naturally infects mice, causes diarrhea and shares 67% of its genes with EPEC and EHEC, including genes associated with pathogenicity and virulence [1]. Cr infection in mice causes attaching and effacing (A/E) lesions and a potent TH1/TH17 inflammatory response similar to those observed in human EPEC/EHEC infections. Therefore, it has become a standard small-animal model to study infectious colitis [8,9]. Cr infection results in various changes to the colon of mice, including epithelial cell proliferation, crypt hyperplasia, an uneven apical enterocyte surface, crypt dilation, and mucosal thickening [10]. Except in highly susceptible mouse strains such as C3H/HeN, colonization of Cr is limited to the colon, with few bacteria reaching systemic organs and the bloodstream [11,12]. Following oral administration, Cr initially colonizes the cecal patch and then migrates to the colon by day 3 post-infection. Bacterial load in the distal colon peaks by day 7 and remains at high levels through day 12, and is typically cleared by day 21 [13]. In general, Cr infection can serve as a useful model for studying compounds that may prevent or mitigate the effects of EPEC or EHEC infections.

**Figure 1:**
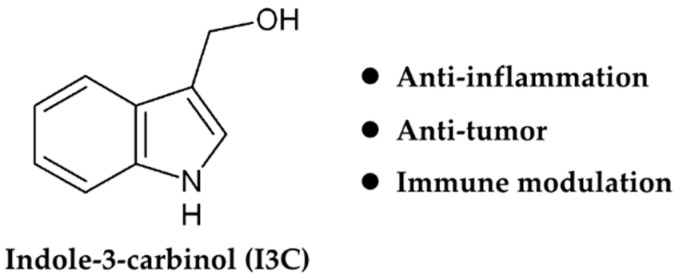
The chemical structure and pharmacological activities of indole-3-carbinol (I3C).

Indole-3-carbinol (I3C) is a dietary compound (Figure 1) derived from glucobrassicin, a glucosinolate found in cruciferous vegetables cruciferous vegetables such as broccoli, cabbage and cauliflower. The concentration of I3C from cruciferous vegetables can be inferred from the content of glucobrassicin which varies in different kinds of cruciferous vegetables, ranging from 0.24 μmol·g^−1^ DW to 6.2 μmol·g^−1^ DW [14,15]. I3C and its derivatives have attracted increased attention due to their important role as anti-inflammatory, anti-tumor and immune modulating agents [16,17]. Substantial evidence indicates that the anti-inflammatory and anti-cancer effects of I3C and its metabolic derivatives are attributed to their ability to modulate several nuclear transcriptional factors including the estrogen receptor (ER), nuclear factor-κB (NF-κB), and the aryl hydrocarbon receptor (AhR), which contribute to maintaining hormonal homeostasis, inhibiting cell cycle progression/apoptosis, inducing DNA repair, and enhancing carcinogen metabolism [17,18]. Although the potential value of I3C and its derivatives in cancer prevention and therapy are well known, the exact underlying mechanisms are still unclear. 

Infection-induced inflammation is a common risk factor for certain types of cancer. Cr infection induces both innate and adaptive immunity, involving the recruitment of immune cells and the release of multiple cytokines and antimicrobial peptides that, are required for clearance of Cr [19,20,21]. It is well known that IL-22 producing CD4^+^ T cells are essential for controlling Cr infection; however, the T cell-derived cytokines (IL17A, IFN-γ and TNF-α) contribute to intestinal tissue injury either directly or indirectly [22,23]. In addition to CD4^+^ T cells, the group 3 innate lymphoid cells (ILC3s) also produce IL22, which is crucial for clearance of Cr [24]. AhR-deficient mice lack IL-22-producing ILC3 in the intestinal lamina propria, and have increased mortality when infected with Cr, suggesting that AhR-dependent signal pathways are important in controlling and clearing Cr [25]. I3C is a powerful AhR activator, which helped suppress Cr-induced inflammation by enhancing AhR activation [26]. Although the protective effect of I3C against Cr infection is known, little information is available related to effects of I3C on the immune response to Cr infection in Cr-susceptible mice. Hence, further study of the mechanism of action of I3C and/or cruciferous vegetable consumption on the immune and inflammatory responses to Cr infection is warranted. 

To address these questions, we focused on characterizing the effects of dietary I3C on the immune response to Cr infection in inflammation-susceptible mice (C3H/HeN strain) to gain further insight into the immunomodulatory properties of dietary I3C. We found the protective effects of dietary I3C against Cr infection occur by down regulating the proinflammatory response while maintaining IL-22 production.

## 2. Materials and Methods

### 2.1. Animals and Diet

C3H/HeN and C57BL/J6 (5-week-old male) mice were purchased from Charles River (Frederick, MD). Mice were housed in ventilated filter-top cages at the USDA BHNRC animal facility under 12-h light/dark cycle. One week of acclimation on chow diet was conducted prior to the dietary treatments. Mice were then, randomized into four experimental groups (n = 8 per group): (1) Uninfected mice on control diet, (2) infected mice on control diet, (3) uninfected mice on treatment diet, and (4) infected mice on treatment diet. Mice were treated for two weeks prior to Cr infection and remained on their respective diets until the end of the experiment. Body weights and food consumption were recorded weekly. All experiments were approved by the USDA-ARS Beltsville Institutional Animal Care and Use Committee (18-027).

Mice were fed an AIN-93M diet with or without 1 µmol I3C/g diet. I3C was purchased from Sigma Chemical Company (St. Louis, MO, USA). A dose of 1 µmol I3C/g diet (147 mg/kg) in mice is roughly equivalent to a dose of 11.4 mg/kg in average adult human. In a clinical study, consumption of I3C was tolerated in doses up to 1200 mg/day in male and female cancer patients. Our selected concentration of I3C for this study was in the range achievable through dietary consumption as well as in a low dose chemo-preventive range [27,28,29].

### 2.2. Cr Infection

Mice were infected with Cr using established protocols [26,30,31]. The Cr strain used in this study was a nalidixic acid-resistant mutant of strain DBS100 (ATCC 51459). A frozen stock of Cr was streaked out on a Luria-Bertani (LB) agar plate and grown overnight at 37 °C. An overnight LB culture grown at 37 °C was started by picking one well-isolated colony. The culture was then expanded and grown to an OD600 nm of ≈1.5, the bacteria were collected and re-suspended in LB medium to a concentration of 1.25 × 10^10^ CFU/mL. After fasting 4–6 h, mice were infected by oral gavage with 0.2 mL of the bacterial suspension (2.5 × 10^9^ CFU). To serve as uninfected controls mice on either diet were given only LB medium. The dose was confirmed by retrospective plating on LB agar plates containing 50 µg/mL nalidixic acid.

### 2.3. Sample Collection

On days 4, 7, 11, 14, 17, and 20 post-infection, fresh fecal pellets of mice were collected for determining the Cr load in feces. The pellets were homogenized in LB broth, serially diluted, and then plated on LB/agar plates containing 50 μg/mL nalidixic acid, incubated at 37 °C overnight. The colonies were enumerated the following day.

Mice were weighed and then euthanized on day 12 and 21 after infection. Blood samples were collected, the serum separated and stored at −80 °C. The spleen, cecum and the distal 5 cm of colon (without fecal pellets) were removed aseptically and weighed. 1cm portions of the distal colon were fixed in 4% formalin for histology or snap frozen in liquid nitrogen for gene expression analysis. The remainder of colon was homogenized and used to measure the tissue Cr load by plating serial dilutions on LB/agar plates containing 50 μg/mL nalidixic acid. Results are expressed as CFUs per gram of colon.

### 2.4. Histological Analysis

Approximately 1 cm sections of distal colon tissue in each group of mice were fixed in 4% formalin and embedded in paraffin, 5-μm sections were cut and stained with hematoxylin and eosin (H&E). The histological grading of coded sections was evaluated for the degree of edema (0–3), surface of epithelium (0–4), loss of crypt architecture (0–4), degree of hemorrhaging (0–4), and the presence of an inflammatory cell infiltrate (0–4). Crypt depth was measured using a Nikon Eclipse E800 microscope and Nikon NIS-Elements software V4.6. Only well-oriented crypts were measured, and 12 or more individual measurements were averaged for each mouse.

### 2.5. Gene Expression Analysis

To determine the gene expression in spleen and colon samples, total RNA was harvested from splenic and colonic tissue using RNeasy Mini kit (Qiagen, Valencia, CA, USA) and TRIzol reagent (Life Technology, NY, USA), respectively. The concentration and integrity of RNA were measured using a Bioanalyzer (Agilent 2100 Bioanalyzer, Santa Clara, CA, USA). RNA with an integrity number above 8 was used for real-time qRT-PCR. The Affinity Script Multi-temperature cDNA Synthesis kit from Agilent was used to reverse-transcribe mRNA to complementary DNA. Real-time PCR was performed on Applied Biosystems ViiA7 Real-Time PCR System using TaqMan^®^ Gene Expression Assay (Invitrogen, Carlsbad, CA, USA). To evaluate the effects of treatment, genes of interest were normalized to the housekeeping gene TATA box binding protein (Tbp) and analyzed using the ∆∆Ct method. The primers/probes for gene expression analysis were purchased from Life Technology are as follow (Table 1):

### 2.6. Serum Cytokines Analysis

Serum cytokines profiles of mice (12 days post-infection) were assessed by using a Bio-Plex Pro™Mouse Cytokine 23-plex assay (Bio-Rad, Hercules, CA, USA) that was performed on a Luminex 200 system and Bioplex HTF in accordance to the manufacturer’s instructions. The results were analyzed using Bio-plex Manager™ software (Bio-Rad, Hercules, CA, USA).

### 2.7. Immunoglobulin Analysis

Nunc Maxi-Sorb plates (Corning, NY, USA) were coated with Cr antigen (10 μg/mL; 50 μL per well in 1X PBS), overnight at 4 °C. After washing with PBS mixed with Tween 20 0.05% (PBS-T), the plates were blocked with 3% nonfat dried milk in D-PBS (100 μL/well) for 2 h at room temperature. Plates were washed 3 times with PBS-T and then 50 μL of mouse serum (12 days post-infection) diluted (1:300 for IgG, M and 1:20 for IgA) in PBS-Tween was added to each well, incubate at 37 °C for 30 min. After incubation, the wells were washed 4X using PBS-T and 50 μL of 1:300 diluted Biotinylated anti-mouse IgG/IgM (Vector Laboratories, Burlingame, CA, USA) was added to each well. After 30 min of incubation at 37 °C, the plates were washed 4X with PBS-T and 50 μL/well streptavidin-HRP (1:1000 dilution from stock) (Vector Laboratories, Burlingame, CA, USA) was added. The plates were then incubated for 30 min at 37 °C, followed by the addition of TMB substrate (preheated at 37 °C, mix before using) for colorimetric detection. The reaction was stopped by adding 50 μL of 4N H_2_SO_4_ and the absorbance at 450 nm was measured immediately using a microtiter plate reader (Molecular Devices, Sunnyvale, CA, USA). Data is expressed as either the OD450 nm or the ratio of infected OD450 nm/uninfected OD450 nm.

### 2.8. Statistcal Analysis

Results are expressed as the mean ± standard deviation (SD). Statistical analysis of this study was conducted by using GraphPad Prism 7 (2018, GraphPad Software, San Diego, CA, USA). Significance of differences between the mean of each group were analyzed using Student’s t test or one-way ANOVA followed by Fisher’s LSD test. In figures where more than two treatment groups are compared, groups with different letter are statistically significantly different (*p* value < 0.05).

## 3. Results

### 3.1. Effect of Cr Infection and I3C Supplementation on Food Intake and Body Weight

**Figure 2:**
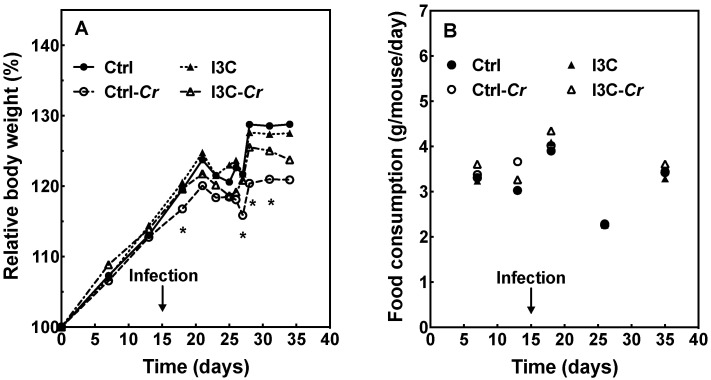
I3C ameliorates the loss of body weight caused by *Citrobacter rodentium* (Cr) infection but has no effect on food consumption in mice. (**A**) Body weight, the body weight of mice on different days was normalized to each animal’s body weight on day 0. (**B**) Food consumption, infection with Cr was initiated on day 15 after start of the diet. Results were expressed as mean or mean +/− SD (n = 8), * indicates significant difference between the Ctrl-Cr and I3C-Cr groups (*p* < 0.05). Only within-day comparisons were made. Ctrl: control diet; Ctrl-Cr: control diet, Cr-infected; I3C: I3C diet; I3C-Cr: I3C diet, Cr-infected.

Figure 2A illustrates the temporal changes in body weight of uninfected and infected C3H/HeN mice fed control or I3C diet. In comparison with the control uninfected group, Cr infection resulted in weight loss in mice fed either diet. Infected mice fed the control diet lost a significant amount of body weight, while the body weight of infected mice on the I3C diet only decreased slightly. The weights of infected mice on the control diet were significantly lower than that of infected mice on the I3C diet at various days’ post-infection (4, 9, 10, 11). Additionally, there was a significant drop in food consumption after the infection (Figure 2B), but it was recovered by the end of the experiment, and there were no significant differences in food consumption between the four experimental groups.

### 3.2. Effects of Dietary I3C on Cr Colonization in Feces and Colon Tissue

**Figure 3:**
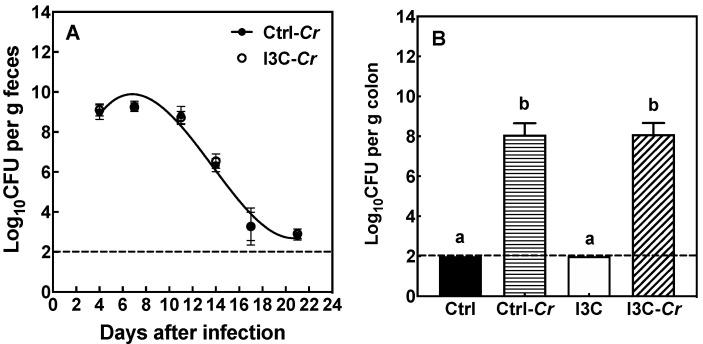
I3C has no impact on Cr burden in feces and colon of Cr-susceptible C3H/HeN mice. Mice were infected orally with approximately 2.5 × 10^9^ CFU of Cr. (**A**): Fecal excretion of Cr. The fecal pellets of mice were collected on different days after infection and the Cr load in feces (cfu/g feces) were determined. (**B**): Colonic colonization of Cr. Mice were sacrificed on day 12-post-infection and the amount of colon tissue associated Cr was determined. Results were expressed as mean +/− SD (n = 8). Ctrl: control diet; Ctrl-Cr: control diet, Cr-infected; I3C: I3C diet; I3C-Cr: I3C diet, Cr-infected. The dashed line indicates the limit of detection of the assay. Significant differences (*p* < 0.05) between groups are identified by different letters.

In both diet groups, mice had significant levels of Cr in the feces by day 4 post-infection that was substantially cleared by day 20 (Figure 3A). There were no differences in fecal Cr load between the two dietary treatments during the infection. Moreover, there were no significant differences in colon Cr colonization on day 12 post-infection between mice fed the control or I3C diet (Figure 3B).

### 3.3. Effects of Dietary I3C on Colon and Cecum Weight in Mice

After oral gavage with Cr, mice develop colitis, leading to a thickened and often shortened colon, and less well-formed stools. As shown in Figure S1, the weight of colon and cecum were significantly increased in infected mice compared to uninfected mice on both days 12 and 21 post-infection. However, there was no effect of I3C on colon and cecum weights when comparing infected groups. 

### 3.4. Effects of Dietary I3C on Histologic Changes in Colon of Mice

**Figure 4:**
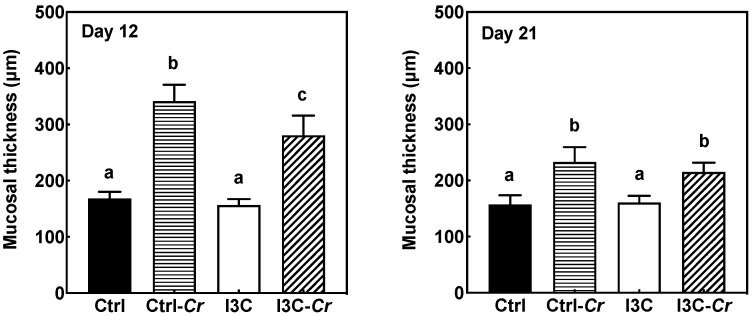
I3C reduces mucosal thickness at day 12 but not day 21 post-infection. Colon tissue was collected on day 12 and day 21 after infection and processed for H&E-staining. Mucosal thickness was measured on well-oriented crypts and 12 or more individual measurements were averaged for each mouse. Results were expressed as mean +/− SD (n = 8). Ctrl: control diet; Ctrl-Cr: control diet, Cr-infected; I3C: I3C diet; I3C-Cr: I3C diet, Cr-infected. Significant differences (*p* < 0.05) between groups are identified by different letters.

Colon tissues obtained on day 12 and day 21 post-infection were processed for H&E staining (Figure S2). The degree of colon pathology in mice on each diet was determined by assessing colon sections. Cr infection resulted in colonic mucosal damage, including increased mucosal thickness, loss of crypt architecture, crypt abscesses, and erosions. Specifically, infected mice fed the I3C diet had significantly lower mucosal thickness on day 12 post-infection compared to the infected mice fed the control diet (Figure 4). On day 21, when the Cr infection was essentially cleared, mucosal thickness in infected mice was reduced compared to day 12. However, there was no significant differences in the mucosal thickness between infected mice fed control and I3C diet on day 21 after infection (Figure 4). In general, there appeared to be little differences in pathology of colon tissues between the diet groups.

### 3.5. Effects of Dietary I3C on AhR and Immune Markers in Colonic Tissues

**Figure 5:**
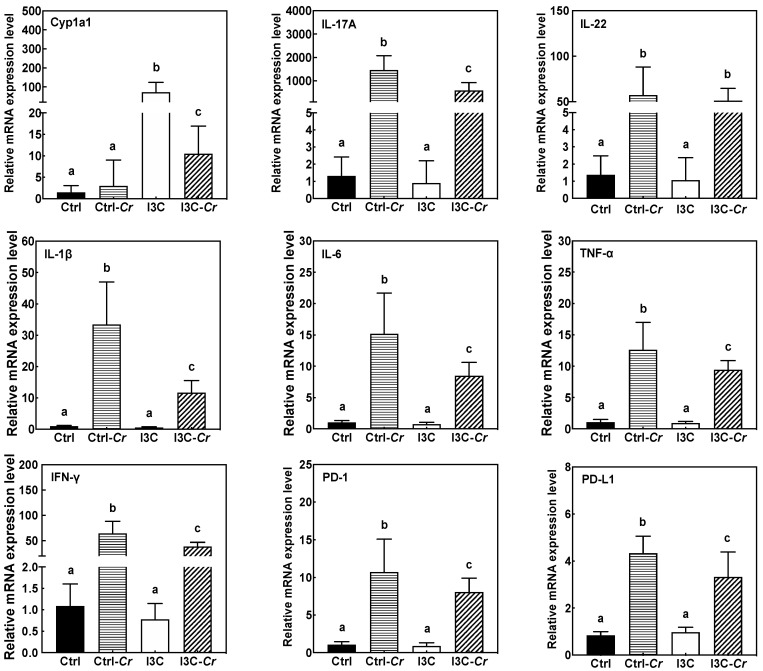
I3C significantly increased xenobiotic metabolizing enzyme genes (Cyp1a1), and attenuated inflammatory markers (IL17A, IL6, IL-1β, TNF-α and IFN-γ) and immune checkpoint inhibitors (PD-1 and PD-L1) gene expression in the colon of infected mice, while it has no effect on Cr-induced increase of IL-22 mRNA. RT-PCR was performed on total RNA isolated from colon tissues harvested on day 12 post-infection. Results are expressed as the mean +/− SD fold-change (n = 8). Ctrl: control diet; Ctrl-Cr: control diet, Cr-infected; I3C: I3C diet; I3C-Cr: I3C diet, Cr-infected. Significant differences (*p* < 0.05) between groups are identified by different letters.

Molecular changes related to AhR and immune pathways were analyzed to help elucidate potential mechanisms of action for I3C (Figure 5). Intake of 1 µmol I3C/g diet significantly induced the expression of the AhR-responsive gene cytochrome P450 1A1 (Cyp1a1) in colonic tissue as compared to animals on control diet. Cr infection significantly attenuated I3C-induced expression of Cyp1a1 mRNA. As expected, mice infected with Cr had increased colonic tissue expression of several cytokines associated with a pro-inflammatory Th1/Th17 immune response including IL-17A, IL-22, IL-6, IL-1β, TNF-α, and IFN-γ on day 12 after infection. Cr-induced expression of IL-17A, IL-6, IL-1β, TNF-α, and IFN-γ mRNA were significantly attenuated in infected mice fed the I3C diet. In contrast, I3C did not affect Cr-induced increase of IL-22 mRNA as compared to control diet. In addition, we also determined gene expression of several markers for CD8+ cell including cd8a, cd8b and FasL. As shown in Figure S3, these markers were upregulated by Cr infection but were not affected by dietary I3C.

### 3.6. Effects of Dietary I3C on Cytokine Levels in Serum

**Figure 6:**
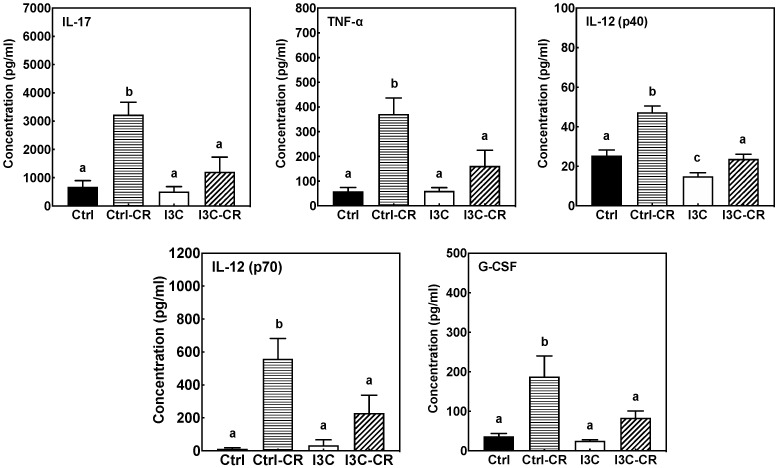
I3C significantly decreased Cr-induced levels of IL17, TNF-α, IL-12, and G-CSF in serum of infected mice fed control diet. Results are expressed as the mean +/− SD (n = 8). Ctrl: control diet; Ctrl-**Cr**: control diet, Cr-infected; I3C: I3C diet; I3C-Cr: I3C diet, Cr-infected. Significant differences (*p* < 0.05) between groups are identified by different letters.

Circulating cytokines levels in mouse serum were also assessed. Cr infection triggered an increase in serum cytokines in Cr-infected mice fed the control diet, especially increasing the levels of IL17, TNF-α, IL12 (p40, p70) and G-CSF (Figure 6). Overall, the levels of serum cytokines in uninfected mice were not affected by diet. More importantly, in comparison to Cr-infected mice fed the control diet, significant reductions in serum levels of IL17, TNF-α, IL12 (p40, p70) and G-CSF were observed in the Cr-infected mice fed the I3C diet, decreasing them to levels near those observed in uninfected mice fed either diet. 

### 3.7. Effects of Dietary I3C on Spleen Size and Immune Cell Molecular Markers

**Figure 7:**
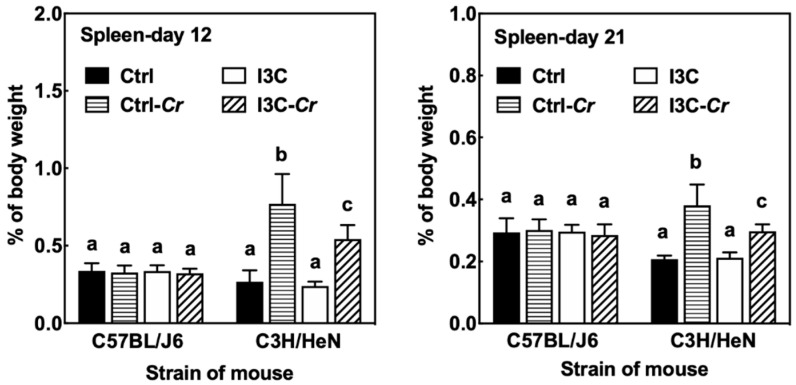
Cr infection resulted in a significant increase in spleen size of C3H/HeN mice while having no effect on the spleen size of C57BL/J6 mice. I3C significantly attenuated Cr-induced increase in spleen size in C3H/HeN mice. Ctrl: control diet; Ctrl-Cr: control diet, Cr-infected; I3C: I3C diet; I3C-Cr: I3C diet, Cr-infected. Significant differences (*p* < 0.05) between groups are identified by different letters.

We found Cr infection led to a significant increase in spleen size only in the Cr sensitive C3H/HeN mice, while Cr infection did not affect the spleen size of Cr-resistant C57BL/J6 mice (Figure 7). Consumption of dietary I3C led to a significant attenuation of the Cr-induced increase in spleen size in C3H/HeN mice. The effect of Cr and I3C on spleen size was still observed even at 21 days post-infection.

**Figure 8:**
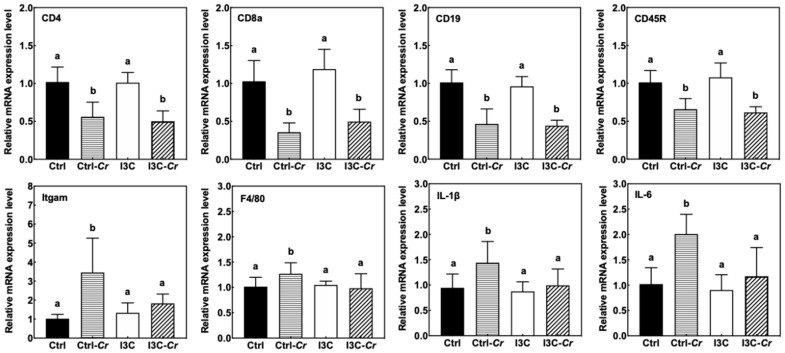
I3C significantly attenuated Cr-induced increases in mRNA levels of macrophage makers (Itgam and F4/80) and proinflammatory cytokines (IL-1β and IL-6) in the spleen. Results are expressed as the mean +/− SD fold-change (n = 8). Ctrl: control diet; Ctrl-Cr: control diet, Cr-infected; I3C: I3C diet; I3C-Cr: I3C diet, Cr-infected. Significant differences (*p* < 0.05) between groups are identified by different letters.

The molecular effects of spleen enlargement were further assessed using specific markers for immune cells (Figure 8). We observed an increase in macrophage makers such as Itgam and F4/80 in the Cr-infected mice. The induction of macrophage markers by Cr-infection was significantly attenuated in the I3C-fed group. Consistent with cytokine expression in the colon, proinflammatory cytokines such as IL-1β and IL-6 were induced by Cr and attenuated by dietary I3C in spleens of Cr-infected mice. However, Cr-infection significantly decreased expression of B- and T-cell markers (CD19, CD45R; CD4, CD8a) in the spleen.

### 3.8. Effects of Dietary I3C on Cr-Specific Antibody Levels in Serum

**Figure 9:**
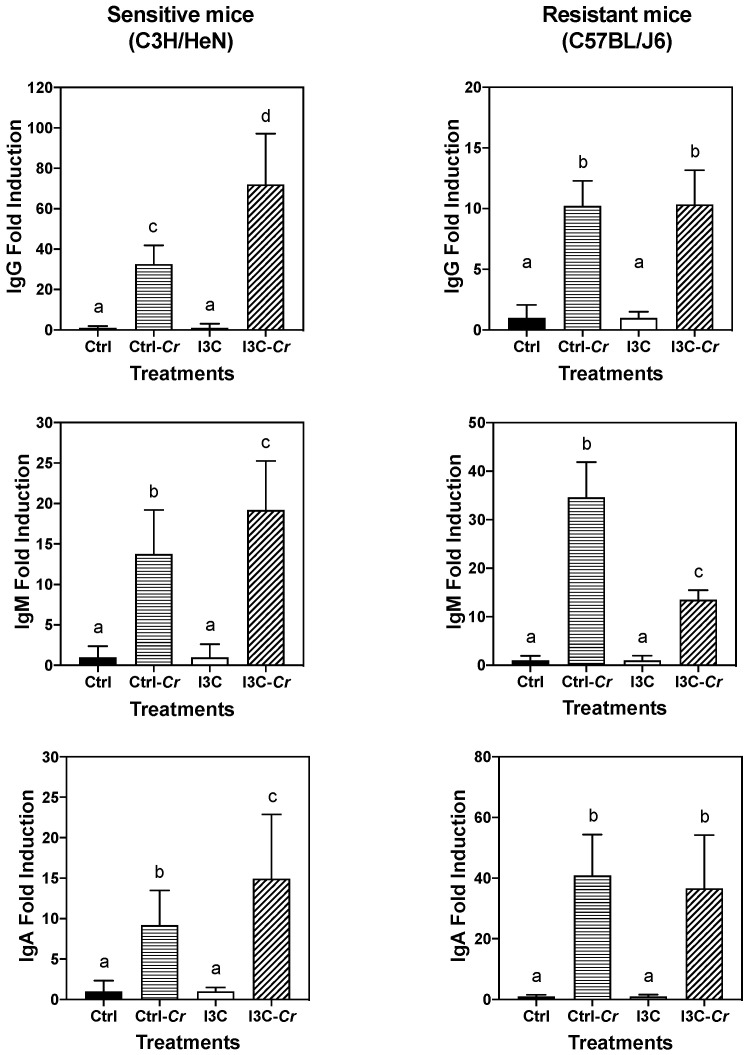
Effects of I3C on Cr-induced IgG response in sensitive (C3H/HeN) and resistant (C57BL/J6) mice. Cr-specific serum antibody levels were calculated as fold change between uninfected controls and infected mice on each diet. Results expressed as the mean +/− SD fold-change (n = 8). Ctrl: control diet; Ctrl-Cr: control diet, Cr-infected; I3C: I3C diet; I3C-Cr: I3C diet, Cr-infected. Significant differences (*p* < 0.05) between groups are identified by different letters.

To further investigate potential mechanisms by which I3C elicits protective effects, we also determined the influence of I3C on Cr-specific serum antibody production by using ELISAs. Considerable variations in antigen-specific IgA, IgG, and IgM production were noted among C57BL/J6 and C3H/HeN mice in each dietary group on day 12 after infection. The data when expressed as OD values (Figure S4) indicates that I3C attenuated the antibody response in resistant C57BL/J6 mice but not in susceptible C3H/HeN mice but similarly reduced the IgM response in the two strains. Serum IgA responses were lower and not affected by diet in either strain. However, it was consistently noted that the background levels of reactivity were lower in I3C treated mice. The reason for this is not clear. But when the data was expressed as fold induction over background dietary I3C treatment led to an enhanced Cr-specific IgG response in Cr-infected C3N/HeN mice but not in C57BL/J6 mice (Figure 9). The effects of Cr infection on Cr-specific IgM and IgA were similar in control- and I3C-fed mice. In contrast, the IgG and IgA responses to Cr infection were similar in Cr-resistant C57BL/6 mice on control and I3C diet, while I3C-fed mice’s IgM responses was significantly attenuated, as compared with that of infected control group.

## 4. Discussion

*E. coli* infections in humans have become an increasing serious global public health issue due to emergent MDR *E. coli* strains [32]. Developing sound strategies and means to inhibit *E. coli* infections would be beneficial for promoting human health and containment of health care cost. I3C derived from cruciferous vegetables, exerting anti-microbial, anti-inflammatory, and anti-tumor properties [17,33], may serve as an alternative to antibiotics for prevention and therapy of *E. coli* infection. In the present study, the effects of dietary I3C consumption on Cr infection of Cr-sensitive C3H/HeN mice was examined as a model for human *E. coli* infections. Our study demonstrated dietary consumption of I3C was able to reduce the pro-inflammatory response to a Cr infection and highlights the potential use of dietary regimes to help protect against a common intestinal infection. The amount of I3C administrated in these studies was in the range achievable through dietary consumption, and therefore, is applicable to humans [29,34].

Mice infected with Cr often develop acute colitis accompanied by an overgrowth of Cr and self-limiting inflammation in murine intestinal lumen [10,35]. Dietary I3C alleviated Cr-induced weight loss and suppressed colonic and splenic inflammation in C3H/HeN mice. However, there were no differences in colon and fecal bacteria load between the Cr-infected mice fed control or I3C diet, suggesting that the main effect of I3C consumption was on modulation of the host immune response rather than a direct effect on clearance of Cr. Our results showed that spleen appeared to be one of the main target organs that benefited from dietary I3C. The spleens of C3H/HeN mice were enlarged due to Cr infection and I3C attenuated the Cr-induced splenomegaly in mice. The spleen enlargement is probably due to an increase in macrophages as indicated by the increase in macrophage marker expression and macrophage-associated cytokines in infected control-fed mice compared to I3C-fed mice. This finding was consistent with Maaser et al. who found that the normally narrow marginal zone of spleen in mice was widened, with numerous macrophages, at 2 weeks after infection [36]. In addition, CD4^+^ T cells and B cells are essential for the development of immunity and clearance of the pathogen [19,22]. We found that Cr-infection appeared to lower expression of T-cell and B-cell markers in the spleen regardless of diet. These data support a depletion of these immune cells in the spleen in response to Cr infection. However, this effect was not modulated by dietary I3C. The decrease of spleen size in Cr-infected mice fed I3C correlates with the reduced levels of circulating pro-inflammatory cytokines and chemokines.

The colon is the primary tissue affected by Cr infection. The infection of mice with Cr induces a robust innate and adaptive mucosal immune responses, and causes various changes to the colon that include epithelial cell proliferation, crypt hyperplasia, crypt dilation, an uneven apical enterocyte surface, and mucosal thickening [22,24,37]. Infected I3C-fed mice appeared to have similar colonic pathology as the infected control diet-fed mice. However, our data showed that dietary I3C significantly reduced the increase in the mucosal thickness in response to Cr infection on day 12 but not on day 21 post-infection. The I3C-induced reduction in mucosal thickness is associated with a decreased pro-inflammatory response in I3C-treated mice at day 12 post-infection. By day 21, the infection is essentially cleared in both control and I3C-treated mice. Substantial healing of the mucosa had occurred by day 21 and no differences mucosal thickness were observed between infected control and I3C-treated mice. 

Most cytokine markers of Cr-induced inflammation were attenuated in colon tissue from Cr-infected mice on I3C diet compared to the infected mice on control diet, suggesting an anti-inflammatory effect of I3C on pathogen-induced acute colitis. The effects appeared to be selective as IL-22 mRNA levels that are closely associated with protection against Cr were unaffected by I3C treatment, while the cytokines associated with pathogenicity, such as IL17A, IL6, IL-1β, TNF-α, and IFN-γ, were decreased by feeding I3C. Furthermore, the anti-inflammatory effects of I3C appeared to be systemic as serum cytokines and chemokines markers (IL17, TNF-α, IL12 (p40, p70) and G-CSF) induced by Cr infection were all significantly attenuated in infected animals fed I3C diet. 

In addition to the suppression of pro-inflammatory cytokines, the protective effects of I3C may be due, at least in part, to maintaining IL-22 levels. IL-22 was demonstrated to be protective against Cr infection and is a potent inducer of antimicrobial peptides, including β-Defensin, Lipocalin-2, RegIIIg and mucins [23,38]. Many types of immune cell, such as CD4^+^ T cell and ILC3 cell, secrete the anti-inflammatory cytokine IL-22 [24,39]. Production of IL-1β and IL-17A and the AhR receptor pathway have been reported to modulate the production of IL-22 [40,41,42]. Additionally, I3C may stimulate IL-22 production via the gut microbiota [43]. Given the systemic inhibitory effects of I3C consumption on IL-1β, IL-17A and its known action as an AhR agonist, it is likely that I3C is acting through AhR to maintain IL-22 expression and while repressing expression of pro-inflammatory cytokines. The precise target cells for I3C will require further validation.

The spleen has two critical functions in host defense against Cr infection, one is removing bacteria from bloodstream, another is producing antibodies for clearance of Cr [44]. A possible mechanism that may explain effect of I3C on spleen and systemic immune responses is related to Cr-driven IgG production. IgG antibodies are required for mediating protective immune against Cr infection [36]. Mice fed I3C seemed to elicit an enhanced Cr-reactive IgG response due to the Cr infection. The enhanced response may aid the removal of bacteria, and thus attenuate inflammation in spleen and the host’s immune system as a whole. The effect of I3C seems to be specific to IgG and not observed for IgM or IgA. Additionally, the effect of Cr-infection on immunoglobulin appeared to be very different between sensitive C3H/HeN mice and the resistant C57BL/J6. The resistant mice elicit relatively small immunoglobulin response upon Cr-infection and I3C-fed animals exhibited lower IgM levels. This observation is consistent with I3C preventing Cr attachment and growth in colonic tissue in resistant C57BL/J6 mice [26]. 

One of our initial hypotheses was that I3C may act through CD8-dependent cytotoxic responses. However, our data indicates otherwise. Cr infection induced up-regulation of CD8^+^ T cell markers such as Cd8a, Cd8b, and FasL, suggesting involvement of CD8-mediated pathways in Cr infection. However, there were no difference in CD8-related markers between control-fed and I3C fed infected mice. Hence, the role of I3C appears relatively specific to its effect on cytokine and chemokine expression. 

## 5. Conclusions

In summary, the data presented here indicate that the cruciferous vegetable-derived compound I3C can provide an overall anti-inflammatory effect on the host. I3C acts by protecting the host against Cr-infection by maintaining IL-22 levels and reducing expression of other pro-inflammatory cytokines, likely through an AhR-dependent pathway. A protective effect on spleen enlargement and attenuated systematic immune response were also observed and may be related to enhanced IgG response in I3C-fed mice. Hence, it is possible that the host response to *E. coli* infection may be modified by consumption of I3C or cruciferous vegetables.

## Figures and Tables

**Table 1:** The list of PCR primers used in this study.

Primers	Catalog Number	Primers	Catalog Number
TBP	Mm00446971_m1	IL-1β	Mm00434228_m1
Cyp1a1	Mm00487217_m1	TNF-α	Mm00443258_m1
IL-17A	Mm00439618_m1	IFN-γ	Mm01168134_m1
IL-22	Mm01226722_g1	Cd8a	Mm01182108_m1
IL-6	Mm00446190_m1	Cd8b	Mm00438116_m1
Foxp3	Mm00475162_m1	FasL	Mm00438864_m1
Cd4	Mm00442754_m1	Cd5	Mm00432417_m1
Cd19	Mm00515420_m1	Cd45	Mm01293577_m1
Itgam	Mm00434455_m1	F4/80	Mm00802529_m1
PD-1	Mm00435532_m1	PD-L1	Mm03048248_m1

## Supplementary Materials

The following are available online at https://www.mdpi.com/2072-6643/12/10/3148/s1, Figure S1. I3C has no impact on increased colon and cecum weight of infected mice. The colon and cecum weight of uninfected and Cr-infected mice fed control and I3C diet were measured on day 12 (A) and 21 (B) post-infection. Results were expressed as mean +/− SD (n = 8). Significant differences (*p* < 0.05) between groups are identified by different letters. Figure S2. Representative images of H&E stained colon sections of uninfected and Cr-infected mice fed control or I3C diet. The colon tissues were harvested on day 12 (a, b, c, d) and day 21(e, f, g, h) post-infection. (a, e): Uninfected mice fed with control diet; (b, f): Infected mice fed with control diet; (c, g): Uninfected mice fed with I3C diet; (d, h): Infected mice fed with I3C diet. The Cr- infected mice fed control diet had crypt hyperplasia, loss of crypt architecture, crypt abscesses and erosions (panel b). I3C, to a certain extent, could ameliorated the colonic damage caused by the infection on day 12. Original magnification × 10. Figure S3. Cr infection significantly increased mRNA levels of CD8^+^ cell markers (Cd8a, Cd8b and FasL), while I3C has no effect on gene expression of these markers in infected mice. RT-PCR was performed on total RNA isolated from colon tissues harvested on day 12 post-infection. Results are expressed as the mean +/− SD fold-change (n = 8). Different letters indicated significant differences between groups for each tissue (*p* < 0.05). Figure S4. Effects of I3C on Cr-triggered IgG response in Sensitive (C3H/HeN mice) and Resistant (C57BL/J6 mice).
