# Supplementary material for: Dietary Indole-3-Carbinol Alleviated Spleen Enlargement, Enhanced IgG Response in C3H/HeN Mice Infected with Citrobacter rodentium
Source: Nutrients. 2020 Oct 15;12(10):3148. doi: 10.3390/nu12103148 (PMC7602481; doi:10.3390/nu12103148)
Supplement: Supplementary file 1 [file nutrients-12-03148-s001.pdf]

Supplementary Materials

Dietary Indole-3-carbinol alleviated spleen enlargement, enhanced IgG response in C3H/HeN mice infected with *Citrobacter rodentium*

Yanbei Wu[1,2], Jing Wang[1], Qiang He[3], Liangli Yu[4], Quynhchi Pham[2], Lumei Cheung[2], Zhi Zhang[4], Young S. Kim[5], Allen D. Smith[2*], Thomas T.Y. Wang[2*]

[1] China-Canada Joint Lab of Food Nutrition and Health (Beijing), Beijing Technology & Business University, Beijing 100048, P. R. China;
[2] Diet, Genomics, and Immunology Laboratory, Beltsville Human Nutrition Research Center, USDA-ARS, Beltsville, MD 20705, USA
[3] College of Biomass Science and Engineering, Sichuan University, Chengdu 610065, P. R. China
[4] Department of Nutrition and Food Science, University of Maryland, College Park, MD 20742, USA
[5] Nutritional Science Research Group, Division of Cancer Prevention, National Cancer Institute, National Institutes of Health, Bethesda, MD 20892, USA

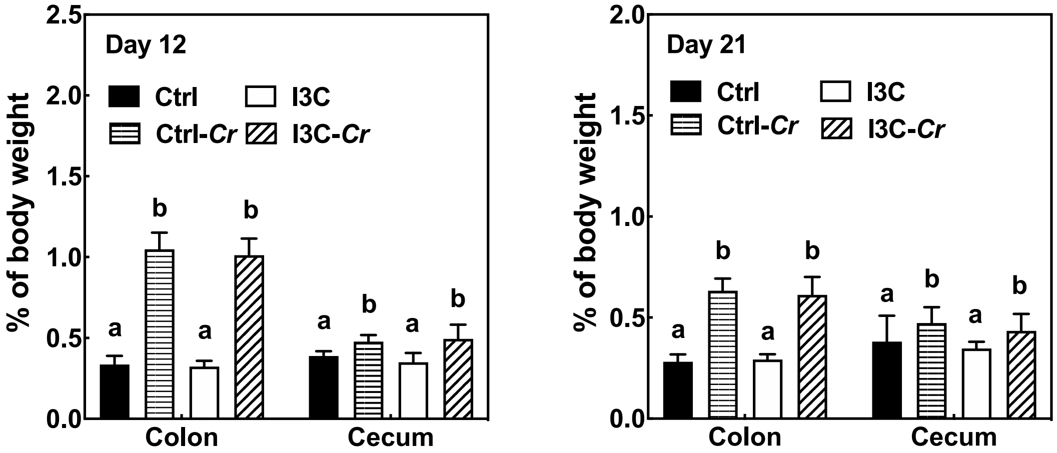

Figure S1. I3C has no impact on increased colon and cecum weight of infected mice. The colon and cecum weight of uninfected and Cr-infected mice fed control and I3C diet were measured on day 12 and 21 post-infection. Results were expressed as mean +/- SD (n=8). Significant differences ($p<0.05$) between groups are identified by different letters.

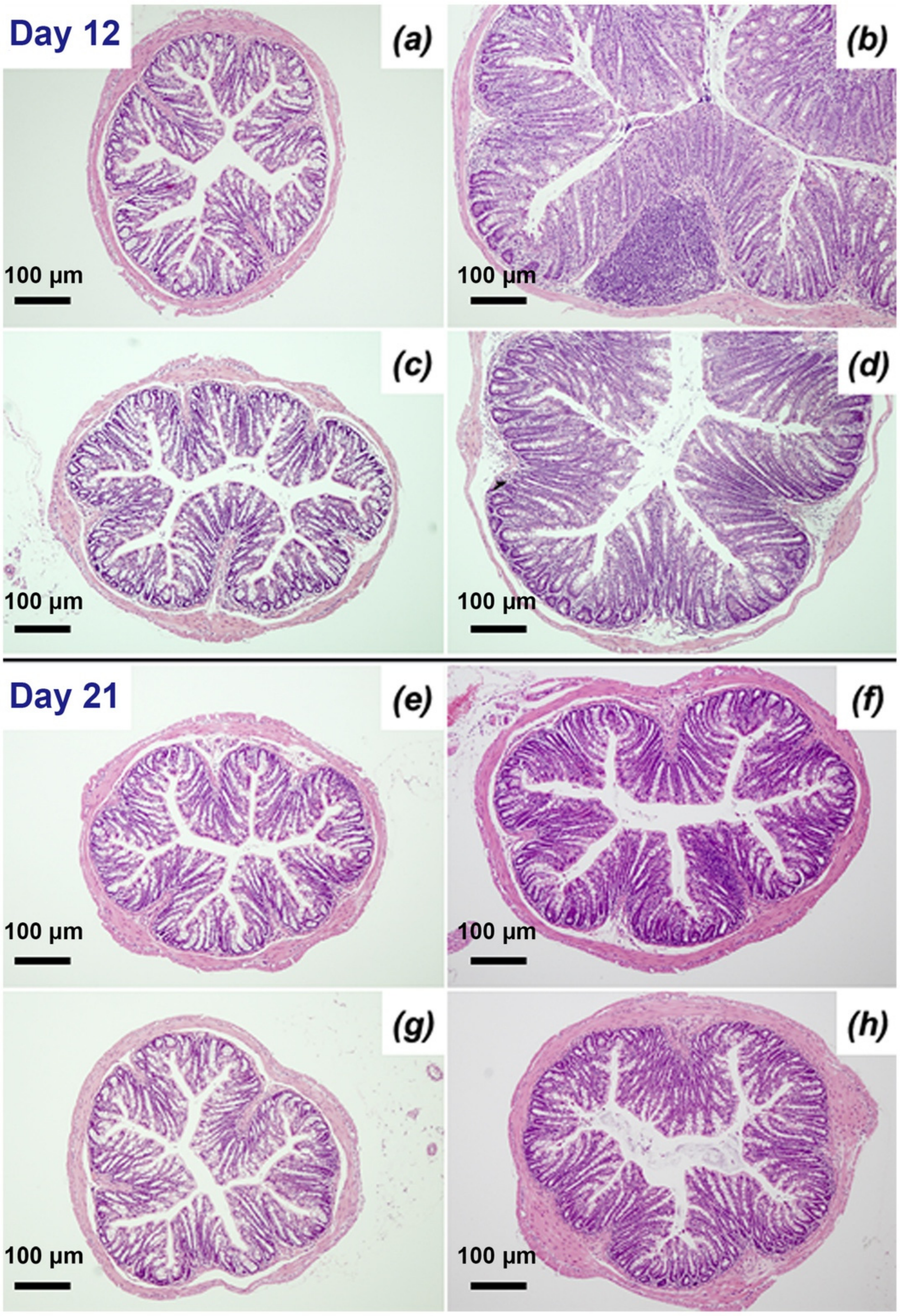

Figure S2. Representative images of H&E stained colon sections of uninfected and Cr-infected mice fed control or I3C diet. The colon tissues were harvested on day 12 (a, b, c, d) and day 21(e, f, g, h) post-infection. (a, e): uninfected mice fed with control diet; (b, f): infected mice fed with control diet; (c, g): uninfected mice fed with I3C diet; (d, h): infected mice fed with I3C diet. The Cr- infected mice fed control diet had crypt hyperplasia, loss of crypt architecture, crypt abscesses and erosions (panel b). I3C, to a certain extent, could ameliorated the colonic damage caused by the infection on day 12. Original magnification × 10, scale bar 100μm.

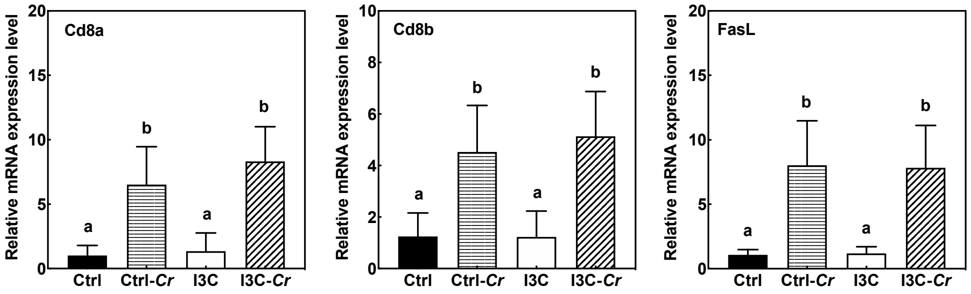

Figure S3. Cr infection significantly increased mRNA levels of CD8⁺ cell markers (Cd8a, Cd8b and FasL), while I3C has no effect on gene expression of these markers in infected mice. RT-PCR was performed on total RNA isolated from colon tissues harvested on day 12 post-infection. Results are expressed as the mean+/-SD fold-change (n=8). Significant differences (p<0.05) between groups are identified by different letters.

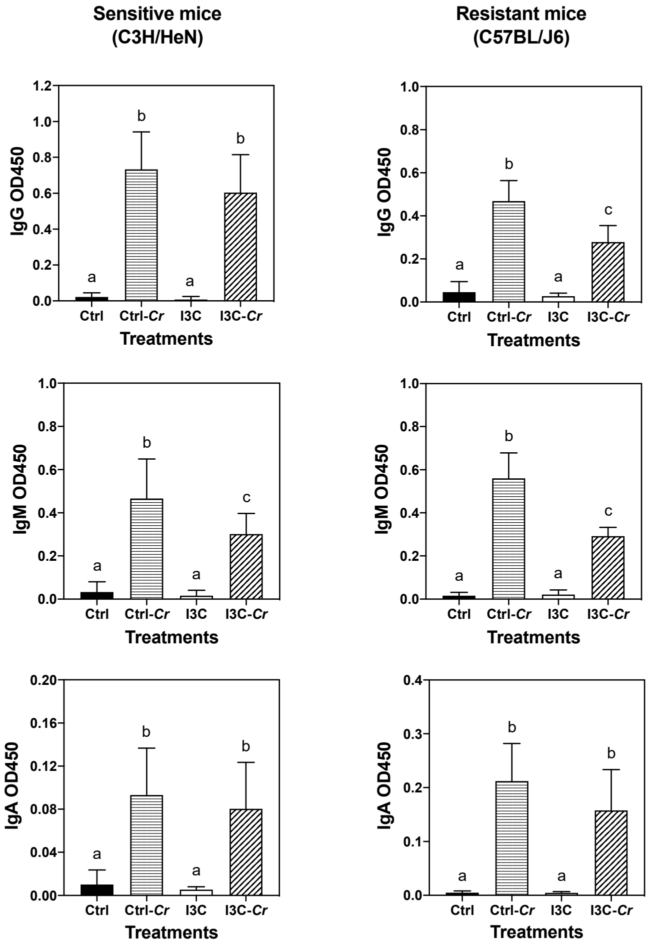

Figure S4. Effects of I3C on Cr-triggered IgG response in Sensitive (C3H/HeN mice) and Resistant (C57BL/J6 mice). Cr-specific serum antibody levels was determined as described in Materials and Methods and the results expressed as OD 450 mean +/-SD (n=8). Ctrl: control diet; Ctrl-Cr: control diet, Cr-infected; I3C: I3C diet; I3C-**Cr**: I3C diet, Cr-infected. Significant differences (p<0.05) between groups are identified by different letters.
